# Coordinate-Corrected and Graph-Convolution-Based Hand Pose Estimation Method

**DOI:** 10.3390/s24227289

**Published:** 2024-11-14

**Authors:** Dang Rong, Feng Gang

**Affiliations:** 1 School of Architecture, Tianjin University, Tianjin 300073, China; fenggangarch@tju.edu.cn; 2 China Construction Engineering Design & Research Institute Co., Ltd., Beijing 100045, China

**Keywords:** hand pose estimation, graph convolutional networks, feature reconstruction, coordinate correction, distributional sensing

## Abstract

To address the problem of low accuracy in joint point estimation in hand pose estimation methods due to the self-similarity of fingers and easy self-obscuration of hand joints, a hand pose estimation method based on coordinate correction and graph convolution is proposed. First, the standard coordinate encoding is improved by generating an unbiased heat map, and the distribution-aware method is used for decoding coordinates to reduce the error in decoding the coordinate encoding of joints. Then, the complex dependency relationship between the joints and the relationship between pixels and joints of the hand are modeled by using graph convolution, and the feature information of the hand joints is enhanced by determining the relationship between the hand joints. Finally, the skeletal constraint loss function is used to impose constraints on the joints, and a natural and undistorted hand skeleton structure is generated. Training tests are conducted on the public gesture interaction dataset STB, and the experimental results show that the method in this paper can reduce errors in hand joint point detection and improve the estimation accuracy.

## 1. Introduction

Human–computer interaction mainly refers to the exchange of information between humans and computers in a certain way to accomplish a certain and defined task. With the continuous advancement of human–computer interaction technology, human–computer interaction is moving from the traditional graphical user interface to a natural human–computer interaction interface; at the same time, the subject of interaction is gradually changing from a computer to a human. Human–computer interaction based on a graphical user interface requires input and output devices such as a mouse, keyboard, and touch screen, and the interaction lacks naturalness, with the computer acting as the main body in the interaction process. With the rapid development of the field of artificial intelligence and the continuous improvement of the required technologies for human–computer interaction, natural human–computer interaction has made remarkable progress, and new types of speech interaction, gesture interaction, etc., have emerged. Natural human–computer interaction is characterized by human subjects so that the computer can understand the user’s intention to interact and perform the corresponding actions, thus improving the efficiency of the interaction.

Of all the parts of the human body, the hand is the most important input device for natural human–computer interaction [1], and gesture interaction is the process of operating a device by tracking the movements of the human hand and translating them into meaningful commands. Gesture interaction has the advantages of ease of use and richness of ideas. With the development of the field of computer vision, gesture interaction based on computer vision is receiving increasing attention and emphasis, and within this field, hand pose estimation based on computer vision is one of the main research focuses [2]. Hand pose estimation is the process of accurately obtaining the coordinates of the key points of the hand from images or videos, inferring the corresponding gestures based on the positional relationship of these coordinates, and achieving non-contact human–computer interaction to improve the user’s interactive experience. Currently, hand pose estimation is widely used in intelligent driving, robot control, VR/AR, and gesture recognition. Due to the frequent hand movements and high degree of freedom, the hand joint points are prone to self-obscuration, which leads to difficulty in estimating the hand joint information.

To address the issue of the low accuracy in joint estimation caused by the self-similarity of fingers and self-occlusion of hand joints in hand pose estimation, this paper proposes a hand pose estimation method based on coordinate correction and graph convolution. First, to alleviate the encoding and decoding errors of hand joint coordinates, a distribution-aware representation of joint coordinates is adopted. Then, a graph convolution module is introduced to construct a graph structure model of the hand joints and enhance the feature information by strengthening the structural relationships between the joints. Finally, a skeleton constraint loss incorporating kinematic principles is utilized to achieve a natural hand structure and effectively improve the accuracy of hand joint estimation. The main contributions of this study are as follows:

(1) When performing hand pose estimation using a stacked hourglass network, it is necessary to generate heatmaps by considering each hand joint coordinate as the mean of a Gaussian distribution. However, this process introduces quantization errors, which, in turn, affect the accurate localization of hand joints. In this paper, we propose the generation of unbiased heatmaps to improve the standard coordinate encoding. Additionally, we adopt a distribution-aware method for coordinate decoding to alleviate the errors in joint coordinate encoding and decoding.
(2) Due to the high degree of freedom in human hands, occlusion often occurs between hand joints, which poses significant challenges for joint prediction. Considering the interdependence among hand joints, we introduce a hand topology structure to extract more features that are beneficial for accurate joint estimation. This allows us to make reasonable predictions for occluded hand joints.
(3) We employ graph convolution to model the complex dependency relationships between hand joints, as well as the relationships between pixels and joints. By determining the relationships between hand joints, we enhance the feature information of each joint. Additionally, we utilize a skeleton constraint loss function to impose constraints on the direction and length of the joints. This approach enables the generation of a natural and undistorted hand skeleton structure.

## 2. Related Work

In recent years, with the development of deep learning and computer vision, there are two main methods for dynamic gesture recognition. One is to directly classify and recognize different gestures by constructing feature-based neural network models. The other is to construct skeleton-based neural network models, which first detect the skeletal joints of the hand and then classify and recognize gestures based on their positions. Gao et al. [3] proposed a dynamic gesture recognition method based on 3D pose estimation. This method uses 3D hand pose estimation, data fusion, and deep neural network technology to improve the recognition accuracy of dynamic gestures. Firstly, improve the 2D pose estimation method based on OpenPose to obtain a fast 3D pose estimation method. Secondly, the weighted and fusion method is used to fuse the RGB, depth, and 3D skeleton data of the gesture. Finally, the 3DCNN+ConvLSTM framework is used to recognize and classify the combined dynamic gesture data. Okano et al. [4] used MediaPipe Hands to detect key points of hands in each frame, which are represented by their three-dimensional coordinates. Then, the recognition accuracy of nine different dynamic gestures was evaluated using the spatiotemporal graph convolutional network (ST-GCN). Zhang et al. [5] proposed a two-stage network model. The first stage is a palm detector, which operates on a complete input image and locates the palm through an oriented hand-bounding box. The second stage is a hand joint point detection model, which takes the hand-bounding box obtained in the first stage as input to obtain the three-dimensional coordinates of each hand joint point. Hakim et al. [6] used 3D convolutional neural network (3D CNN) and long short-term memory (LSTM) as feature extraction models. Using multimodal data, RGB, and depth data as input models, finite state machine (FSM) control is used to restrict certain gesture flows and recognition classes.

At the same time, in order to better study dynamic gesture recognition based on neural networks, a large number of dynamic gesture recognition datasets have been proposed. Materzynska et al. [7] proposed a large-scale gesture recognition real-world video dataset, Jester. This dataset contains 144,892 video clips of 3 s in length, accounting for over 5 million frames, and collects 27 gesture actions from 1376 participants. Gupta et al. [8] proposed a multimodal dynamic gesture dataset, NVIDIA, which was captured by depth, color, and stereo infrared sensors and collected 1532 dynamic gestures, including 25 gesture types. Benitez Garcia et al. [9] proposed the IPN Hand dataset, which contains over 4000 gesture samples and 800,000 RGB frames from 50 different topics, including 13 different static and dynamic gestures for non-touch screen interaction.

Through reading the literature on dynamic gesture recognition, it was found that the accuracy of hand joint detection can improve the accuracy of gesture recognition. Therefore, in recent years, there have been a large number of studies on hand joint detection, namely hand pose estimation. These algorithms can be categorized into two types based on the input data: depth-based and color-based. Depth-based hand pose estimation algorithms require the use of depth cameras for data acquisition and are limited to indoor usage. Furthermore, they are expensive and come with restrictions on their usage scenarios and range. On the other hand, color-based hand pose estimation only requires regular cameras, making it more widely usable in various daily life scenarios. Therefore, research on color-based hand pose estimation holds greater practical significance.

In the domain of hand pose estimation based on RGB-D images, the various approaches can be classified according to the input information, including those based on regular depth maps, multi-view depth maps, voxel-based methods, and point-cloud-based methods. Mei-Ying Ng et al. [10] employed a self-attention mechanism to enhance convolutional features, capturing long-term dependency relationships in-depth images. They extended the anchor points to the depth dimension to estimate 3D hand joint positions, thereby improving the accuracy of the hand pose estimation. Ge Liuhao et al. [11] projected a depth image onto three orthogonal planes and used convolution to obtain 2D heatmaps from these multi-view projections. They estimated the joint positions on each plane and then fused these multi-view heatmaps together to generate the final 3D hand pose estimation using a learned prior pose. Fang Linpu et al. [12] proposed the JGR-P2O algorithm, which introduces a joint graph determination module based on graph convolutional networks (GCNs). This module models the dependency relationships between joints using a graph structure and performs voting mapping between pixels and joints to enhance the local feature representation learning. Additionally, the algorithm employs pixel-level offset predictions and direct joint regression for end-to-end training of the model. Moon Gyeongsik et al. [13] proposed the V2V-PoseNet algorithm to address the issues of perspective distortion in 2D depth maps and the non-linear mapping challenge of estimating 3D coordinates from 2D images. They introduced the transformation of individual depth maps into a 3D voxel representation, using voxels as the input for the model. Point cloud data consist of a series of three-dimensional spatial coordinates on the surface of the target object. They contain more information than regular depth images and can utilize the 3D information in-depth images more effectively. Therefore, point cloud data can represent the true pose of gestures more accurately. Ge Liuhao et al. [14] proposed the PointNet network model, which first segments the gesture region in the depth image, converts the segmented hand depth image into point cloud data, and performs data normalization and standardization. Then, it directly recreates and estimates the 3D coordinates of hand joints from the 3D point cloud data. Jia Gong et al. [15] introduced the MATAL framework to actively select and label informative images for effective learning. MATAL formulates the image selection process as a Markov decision process and learns the optimal sampling strategy that maximizes the performance of the pose estimator based on rewards, reducing the dataset annotation time. MURAD ALMADANI et al. [16] employed multi-modal inputs, combining 3D representation (i.e., voxelated RGBD) and 2D representation (i.e., RGB images) to effectively handle the complex structures, dynamic variations in hands and objects, and their interactions. Chang et al. [17] proposed a structure-aware symbolic distance for the representation function S-SDF of 3D hands and a self-supervised appearance synthesis method based on monocular RGB images to reconstruct hand surfaces without resolution and topology constraints. Yu et al. [18] improved the accuracy and stability of reconstruction by automatically learning about and focusing on important features of hands through the introduction of attention mechanisms.

The research on hand pose estimation based on color images can be divided into 2D and 3D hand pose estimation based on the form of the output data. Two-dimensional hand pose estimation involves obtaining the two-dimensional position coordinates of key hand points, while three-dimensional hand pose estimation involves obtaining the depth information of key points based on the two-dimensional position coordinates, thus obtaining the three-dimensional spatial position coordinates of the key points. Zimmermann et al. [19] proposed a three-stage cascaded hand pose estimation network, which consists of the HandSegNet, PoseNet, and PosePrior networks arranged in sequence. The output of the previous network serves as the input of the next network. The HandSegNet network is used to perform hand region segmentation in an image, resulting in a hand mask size of 256 × 256 after cropping and resizing. This mask is then fed into the PoseNet network for feature extraction and inference of the positions of 21 key hand points, generating a heatmap of positions. Finally, using the PosePrior and Viewpoint-Combination networks, the position estimates in the 2D heatmap are transformed into the 3D hand pose. The training process of this model is complex. Moon et al. [20] proposed the InterNet model for multi-object hand pose estimation. The InterNet model utilizes ResNet-50 as its backbone network to extract global features from the input data. It applies the PoseNet network, along with dual upsampling and downsampling structures, to process the feature maps of the first stage and infer the heatmaps of two target key hand points. Finally, through fully connected layers, the model determines the number of hands and their left–right types in the image and estimates the Euclidean distance between the main connection points of the two hands. Shreyas Hampali et al. [21] proposed a hand pose estimation method based on the Transformer. This method first uses the U-Net network to obtain the two-dimensional positions of each key hand point and encode them. Then, the encoded image feature is fed into the “Keypoint Transformer” structure, which resolves ambiguities between key points and addresses hand–hand and hand–object interactions. Doosti Bardia et al. [22] introduced the HOPE-Net model based on graph convolution. This model employs ResNet-10 as its image encoder to predict the initial two-dimensional coordinates of joints and object vertices. Subsequently, the coordinates of object vertices and key hand point coordinates are utilized as inputs for a three-layer graph convolution to estimate improved two-dimensional poses based on neighboring features. Finally, the previously predicted two-dimensional coordinates are passed to an adaptive graph U-Net to obtain the three-dimensional coordinates of the hand and object. This method achieves superior pose estimation by constructing a hand topology structure. Ge Liuhao et al. [23] utilized graph convolution to reconstruct the three-dimensional mesh of hand vertices. They extracted features from a regular convolutional neural network and output the three-dimensional coordinates of the mesh vertices through two upsampling layers and four graph convolution layers. The hand pose estimation was accomplished by means of linear regression on the mesh vertices. Leyla Khaleghi et al. [24] proposed the MuViHandNet model, which consists of an image encoder, a temporal learner (LSTMt), an angle learner (LSTMv), and a Graph U-Net. By incorporating recurrent learning of temporal and angle sequence information, the model estimates the 3D pose of a hand using the Graph U-Net. This method effectively utilizes the abundant hand information conveyed by videos and multiple viewpoints.

It is evident that existing methods aiming to improve the accuracy of hand pose estimation have designed complex network models while overlooking the errors introduced by the coordinate transformation of hand joint positions and the interdependencies among these joints. To address these issues, this paper proposes a hand pose estimation method based on coordinate correction and graph convolution. The method utilizes an hourglass network as its base model. It employs a pixel-to-joint voting mechanism to achieve feature mapping from global features to hand joint features. Then, graph convolution is utilized to reconstruct the joint information and enrich the joint features. Next, a distribution-aware coordinate representation is employed to correct the coordinate errors and enhance the accuracy of the hand pose estimation. Finally, a skeleton constraint loss is introduced to achieve natural hand poses.

## 3. Methodology

### 3.1. Overall Network Structure

**Figure 1:**
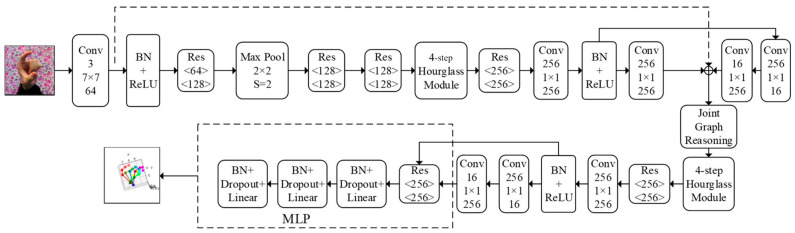
A global network model for hand pose estimation.

The overall network structure of our proposed method is illustrated in Figure 1. This network model primarily consists of two stacked four-stage hourglasses, graph convolution modules, and a multilayer perceptron. The hourglass network utilizes upsampling and downsampling modules to fuse features at different levels, capturing both the local and global information of hand joint positions. This aids in accurately locating the positions of different hand joints and predicting the heatmaps of joint scores. During the training process, these predicted heatmaps are compared with the ground truth heatmaps, serving as intermediate supervision signals to optimize the network model. Additionally, the dense connection module enhances the feature propagation and reuse. The graph-convolution-based graph reasoning module incorporates the physical structure of the hand skeleton into the network. It learns the global contextual information encoded in the local features to generate joint features. Then, through graph reasoning, it further enhances the network’s ability to represent the hand joint positions and improves its local prediction ability. Due to the high degree of freedom in hand movements, occlusion between hand joints can pose significant challenges to joint prediction. Considering the interdependency among hand joints, we introduce hand topology to extract more favorable features for joint positions and make reasonable predictions for occluded hand joints. In the graph reasoning module, several steps are taken to enhance the network’s ability to represent hand joint features. Firstly, a pixel–joint voting mechanism is employed to aggregate the weighted average of local features based on global contextual information, generating hand joint features. Secondly, a node–node undirected graph, G=<N,E>, is defined, where N is the set of individual joints and E is the set of relationships between pairs of joints. This graph structure is used to propagate joint features, capturing the interdependencies between joints and further enhancing the ability to represent the joints. Lastly, a mapping mechanism is employed to map the enhanced joint features back to local features using the joint–pixel mapping and these enhanced features are combined with the original local features to strengthen their representation. An MLP network is utilized to predict the depth information of the hand joints, which is then fused with the 2D joint information to obtain the final 3D coordinates of the hand joints.

### 3.2. Hourglass Network

**Figure 2:**
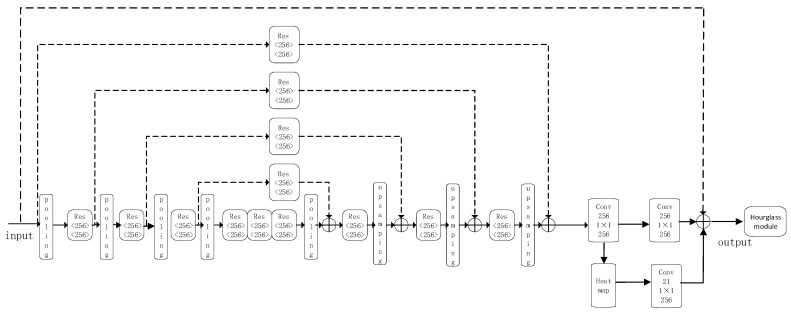
Hourglass network model.

The stacked hourglass network is a classic network model in the field of pose estimation [25]. It comprises a series of individual hourglass networks, where each hourglass network resembles an hourglass shape with symmetric left and right sides. The key feature of the hourglass network is its ability to capture information at different scales by utilizing upsampling and skipping connections, preventing the loss of information. The structure of the hourglass network is illustrated in Figure 2. In the stacked hourglass network, the second hourglass network not only takes the output feature maps from the previous hourglass network as input but also incorporates the heatmaps generated by the previous hourglass network. This helps guide the localization of the key points. Additionally, loss calculation is performed on the predicted heatmaps generated by each sub-hourglass in the stacked hourglass network. All heatmap losses are included in the overall model loss calculation, enabling intermediate supervision and optimizing the network model.

The hourglass network consists of an encoder and a decoder. The encoder utilizes convolutional layers and pooling layers to extract features and downsample the input image, resulting in multi-scale feature maps. The decoder upsamples the feature maps at the lowest resolution to obtain higher-resolution feature maps, which are then fused with the encoder’s output feature maps of the same scale to obtain more accurate features. The internal structure of the hourglass network includes downsampling modules, upsampling modules, and residual modules. The downsampling module consists of two convolutional layers with a stride of 2 and a ReLU activation function. It is used to downsample the input feature maps to a smaller size and extract more abstract high-level features. The upsampling module consists of two transposed convolutional layers with a stride of 2 and a ReLU activation function. It is used to upsample the feature maps from the downsampling module back to the original size and restore the positional information. The residual module consists of two convolutional layers with ReLU activation functions. The first convolutional layer performs feature transformation on the input, while the second convolutional layer maps the features back to the original dimension.

**Figure 3:**
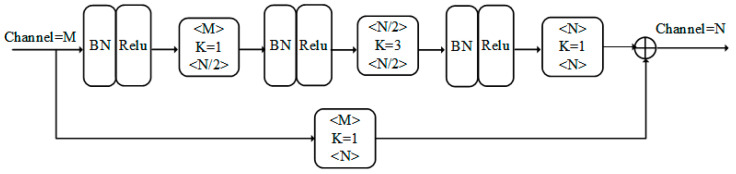
Residual block module.

The primary module of the hourglass network is the residual block, as shown in Figure 3. The residual block can be seen as a structure with skip connections, allowing the neural network to directly learn the residual of the input data and avoiding the loss of information caused by an increasing network depth. Using residual blocks effectively reduces the training difficulty of the neural network while ensuring the model’s performance. The residual block consists of two branches: the upper branch, which is composed of convolution, batch normalization, and activation functions, serves as the feature extraction part, while the lower branch is the skip connection, which is used to preserve the original feature information. The final output of the residual block is the fusion of the features from both branches. This module only changes the number of feature channels and does not affect the feature size.

### 3.3. Coordinate-Corrected and Graph-Convolution-Based Hand Pose Estimation Method

When using the stacked hourglass network for hand pose estimation, it is necessary to generate heatmaps by using the coordinates of each hand joint as the mean of a Gaussian distribution. This process is called coordinate encoding. Compared with regular feature maps, heatmaps can provide more spatial information about hand joints, which is beneficial for accurately locating the positions of hand joints using the network model. However, the process of heatmap generation introduces quantization errors. Additionally, the heatmaps generated by the stacked hourglass network have low resolutions, which increases the amplification of quantization errors and consequently affects the accurate localization of hand joints. Two-dimensional pose estimation requires estimating the coordinates of joints from heatmaps, a process called coordinate decoding. In the existing coordinate decoding process, the coordinates of the maximum activation position on the heatmap are usually selected as the final result, or a manual shifting operation is performed from the maximum activation position towards the second-highest activation position for correction [26]. However, this shifting operation also introduces errors.

The accumulation of errors in the coordinate encoding and decoding processes ultimately leads to errors in the coordinates of hand joints, resulting in inaccurate hand pose estimation. Therefore, this study adopts a coordinate representation method based on distribution awareness [27]. This method consists of two steps: generating unbiased heatmaps centered around non-quantized coordinates during the encoding process and performing coordinate decoding based on Taylor expansion by considering the distribution information of heatmap activations during the decoding process. The coordinate representation method based on distribution awareness is simple and convenient to use, does not affect the network structure and model size, and effectively addresses the limitations of traditional coordinate encoding and decoding for hand joints. By employing this method, more accurate hand poses can be obtained.

#### 3.3.1. Encoding of Hand Joint Coordinates

Let g=(u,v) represent the true coordinates of the hand joints in the original image. In the data preprocessing stage, it is necessary to first resize the original image to match the size of the model’s input. Similarly, the coordinates of hand joints also need to be scaled accordingly. Then, the scaled coordinates of the hand joints are used to generate the corresponding heatmaps, indicating the required transformation of the true coordinates of hand joints during the encoding process. The coordinate transformation process is defined by Equation (1), where λ represents the downsampling rate. In this study, the unquantized coordinate $g^{\prime }=(u^{\prime },v^{\prime })$ is utilized as the center to generate unbiased heatmaps, as shown in Equation (2).

(1) $$g^{\prime }=(u^{\prime },v^{\prime })=\frac{g}{\lambda }=(\frac{u}{\lambda },\frac{v}{\lambda })$$

(2) $$G(x,y;g^{\prime })=\frac{1}{2\pi \sigma^{2}}\exp(-\frac{{\left(x-u^{\prime }\right)}^{2}+{\left(y-v^{\prime }\right)}^{2}}{2\sigma^{2}})$$

#### 3.3.2. Decoding of Hand Joint Point Coordinates

Firstly, assuming that the heat map predicted by the convolutional network model conforms to the standard Gaussian distribution, the predicted heat map can be expressed as Equation (3), where x is the positional coordinate of any pixel in the predicted heat map, μ is the predicted positional coordinate of the hand joint point, and ∑ is the variance matrix.

(3) $$G(x;\mu ,\sum )=\frac{1}{(2\pi ){\left|\sum \right|}^{\frac{1}{2}}}\exp(-\frac{1}{2}{\left(x-\mu \right)}^{T}\sum^{-1}(x-\mu ))$$

In order to efficiently reduce the difficulty of the solution, the exponential form G is first converted to a logarithmic form, as shown in Equation (4):

(4) $$P(x;\mu ,\sum )=\ln(G)=-\ln(2\pi )-\frac{1}{2}\ln(\left|\sum \right|)-\frac{1}{2}{\left(x-\mu \right)}^{T}\sum^{-1}(x-\mu )$$

Since μ is an extreme point in the two-dimensional Gaussian distribution, the first-order partial derivative at μ satisfies Equation (5):

(5) $$D^{\prime }(x){|}_{x=\mu }=\frac{\partial P^{T}}{\partial x}{|}_{x=\mu }=-\sum^{-1}\left(x-\mu \right){|}_{x=\mu }=0$$

P(μ) can be approximated by predicting the Taylor series at the maximum activation m of the heat map, as shown in Equation (6):

(6) $$P(\mu )=P(m)+D^{\prime }(m)(\mu -m)+\frac{1}{2}{\left(\mu -m\right)}^{T}D^{\prime \prime }(m)(\mu -m)$$

where $D^{\prime \prime }(m)$ is the second-order partial derivative of P(μ) at m, as shown in Equation (7):

(7) $$D^{\prime \prime }(m)=D^{\prime \prime }(x){|}_{x=m}=-\sum^{-1}$$

Finally, the hand joint point coordinates in the original image space are obtained using Equation (8):

(8) $$\mu =m-{\left(D^{\prime \prime }(m)\right)}^{-1}D^{\prime }(m)$$

Compared with the standard method that only considers the first and second maximal activations in the heat map, the coordinate decoding method based on Taylor’s theorem fully exploits the rich distributional statistical information in the heat map and can more accurately infer the potential maxima in the heat map through function approximation to obtain more accurate coordinates of joint points of the hand. The coordinate decoding method based on Taylor’s expansion not only has a simple and efficient computation process that only needs to calculate the first-order and second-order partial derivatives of the location of the maximum activation value in the heat map to obtain the accurate coordinates of the joints, but it also does not affect the network model.

### 3.4. Hand Arthrogram Reasoning Module

**Figure 4:**
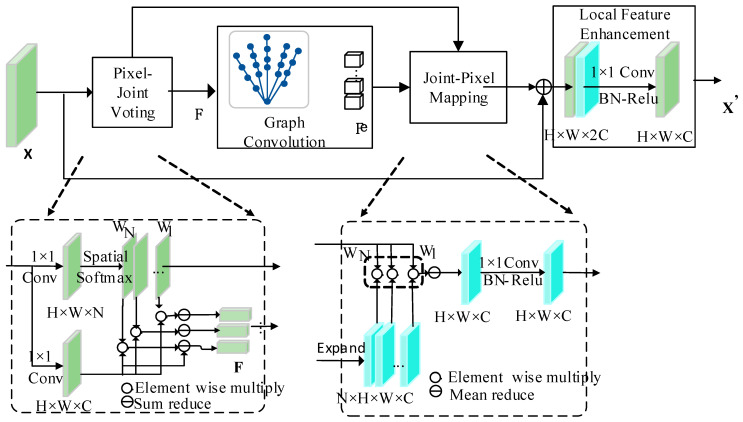
Joint graph reasoning module.

Our hand skeleton is a natural graph structure and thus able to enhance the features of the hand joint points using a graph convolutional network (GCN). The joint graph reasoning (JGR) module obtains richer feature information for each hand joint point by building the hand skeleton and modeling the pixel-to-joint point mapping relationship [12]. The structure of the graph-convolution-based hand joint point map determination module is shown in Figure 4. The input is the intermediate feature X, extracted from the backbone network, and firstly, X generates the feature representation F of the hand joints through the pixel–joint point voting mechanism. Then, the hand joint point features F undergo relational inference and node feature propagation in the graph space to obtain the enhanced hand joint features $F^{e}$. Finally, the enhanced hand joint features are mapped back to the local features through the node–pixel mapping mechanism and fused with the original features X to obtain the final locally enhanced features $X^{\prime }$.

#### 3.4.1. Pixel–Joint Voting

Assuming that the feature map behind the backbone network is $X\in R^{H\times W\times C}$, where H,W,C denote the height, width, and number of channels of the feature map, respectively, the voting weights from pixels to hand joints are calculated as shown in Equation (9):

(9) W=ϕ(φ(X))

where φ(⋅) is the convolutional transform function, ϕ is the spatial softmax normalization, and $W\in R^{H\times W\times N}$ is the voting tensor.

$F_{k}$ is the feature of the kth hand joint, and the feature of joint k is the weighted average of all pixel features, which is calculated as shown in Equation (10):

(10) $$F_{k}=\sum_{i}w_{ki}\psi (X_{i})$$

where $X_{i}$ is the input feature map, ψ(⋅) is the convolutional transform function, $W_{k}\in R^{H\times W}$ denotes the voting matrix of the hand joints k, and $w_{ki}$ is an element of $w_{k}$.

The characteristics of the 21 hand joint points can be expressed as shown in Equation (11):

(11) $$F=\left[f_{1}^{T}\cdot \cdot \cdot f_{21}^{T}\right]$$

#### 3.4.2. Graph Convolution

In the graph convolution operation, each node interacts with its neighboring nodes and computes a new feature vector that represents the performance of that node in each feature dimension. Specifically, the output feature of each node is composed of a weighted sum of its own feature vector and the feature vectors of its surrounding nodes. The weights here are determined by the relationships between nodes, and usually, the neighborhood matrix or degree matrix is used to describe the connections between nodes.

Firstly, the joint-to-joint undirected graph, G=<N,E>, is defined, the features of each joint are input, the dependencies between the joints are modeled using GCN, and the matrix multiplication is used to operate on all the joint features F to obtain the enhanced feature $F^{e}$. The inference process is shown in Equation (12):

(12) $$F^{e}=\sigma (A^{e}FW^{e})$$

where $W^{e}\in R^{C\times C}$ is the transformation matrix obtained from training, and $A^{e}\in R^{N\times N}$ is the connection weight matrix defined in E based on edge connections.

#### 3.4.3. Joint–Pixel Mapping

The mapping of hand joint points to pixels is carried out using the inverse operation of pixel to node. Firstly, the features of hand joints k are calculated as shown in Equation (13), and for pixel $P_{ik}$, the final features can be defined as shown in Equation (14):

(13) $$P_{ik}=w_{ik}f_{k}^{e}$$

where ρ(.) is the convolutional transform function, and N is the number of joints.

### 3.5. Skeletal Constraint Loss Function

**Figure 5:**
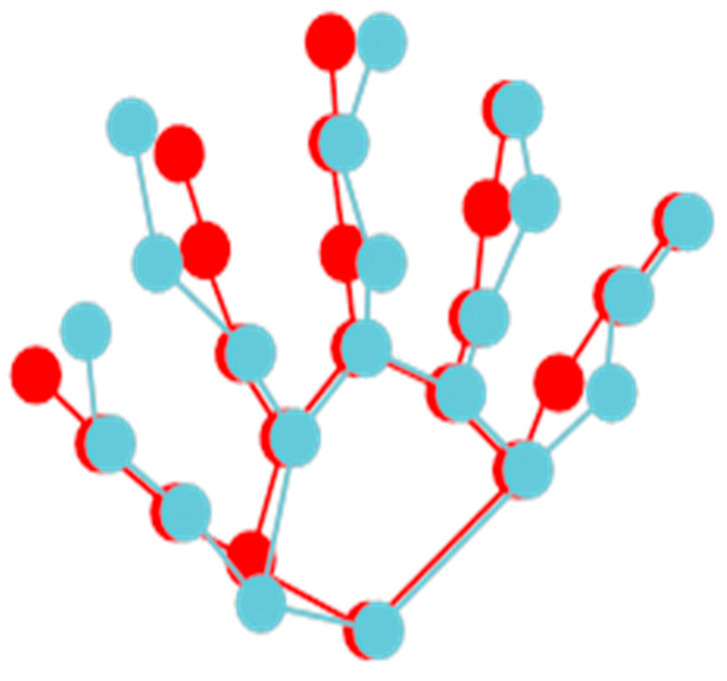
Skeletal topology of the hand.

In 3D hand pose estimation tasks, the loss usually consists of two components: 2D and 3D pose estimation loss. However, this type of loss does not take the constraint relationships between hand joints into account. From the principles of hand kinematics, it can be observed that the human hand is not completely unrestricted. To address this limitation, the hand skeleton constraint loss function, inspired by biology and based on the principles of hand kinematics, applies structural constraints to the estimated hand pose and introduces prior knowledge of hand structure to assist in model training. The main idea is to restrict the joint positions based on the topology of the hand skeleton, leading to a more accurate estimation of hand joint positions and ensuring the generation of a natural and undistorted hand skeletal structure. In order to better explain the difference between the true and predicted value, a visualization of a skeletal joint point is illustrated in Figure 5, where the red color represents the ground truth, and the blue color represents the predicted values.

The steps for implementing the loss of skeletal constraints in the hand are as follows:

Step 1: Determine the skeletal topology of the hand, i.e., how and in what order the palm, phalanges, knuckles, etc., are connected to each other.

Step 2: For each sample in the training dataset, the true position and orientation of the hand bones can be calculated based on the hand bone topology.

Step 3: During model training, the previously computed information about the true position and orientation of the hand bones can be used as the target values for the constraint loss of the hand bones.

Step 4: The hand pose estimation loss function and the hand bone constraint loss function are weighted and summed, and the parameters of the neural network are updated using the back propagation algorithm. In the process of model training, the hand pose estimation and skeletal constraint loss can be optimized at the same time in order to obtain more accurate prediction results. The specific formulation of the skeletal constraint loss is shown below:

(14) $$L_{prop}=\lambda_{hm}L_{hm}+\lambda_{pose}L_{pose}+\lambda_{len}L_{len}+\lambda_{dir}L_{dir}$$

where $L_{hm}$ is the heat map loss for 2D hand pose estimation, $L_{pose}$ is the 3D loss for 3D hand pose estimation, $L_{len}$ is the skeletal length distance loss, $L_{dir}$ is the skeletal orientation loss, and $\lambda_{hm}$, $\lambda_{pose}$, $\lambda_{len}$, and $\lambda_{dir}$ are the weight hyperparameters for each type of loss, which take the values of 0.1, 1, 0.01, and 0.1, respectively. The skeletal length distance loss, $L_{hm}$, and the skeletal orientation loss, $L_{dir}$, are defined in Equations (18) and (19).

The heat map loss, $L_{hm}$, for 2D hand pose estimation is calculated using the mean square error loss, defined in the following equation:

(15) $$L_{hm}={\sum_{j=1}^{21}\left\|h_{j}-{\overset{\wedge }{h}}_{j}\right\|}_{2}^{2}$$

where $h_{j}$ is the true thermogram, ${\overset{\wedge }{h}}_{j}$ is the estimated thermogram, and $L_{hm}$ is the sum of the errors at the 21 hand joint points.

The 3D loss for 3D hand pose estimation, $L_{pose}$, is computed using the mean square error loss, defined in the following equation:

(16) $$L_{pose}={\sum_{j=1}^{21}\left\|\phi_{j}-{\overset{\wedge }{\phi }}_{j}\right\|}_{2}^{2}$$

where $\phi_{j}$ and ${\overset{\wedge }{\phi }}_{j}$ are the real and estimated 3D joint point coordinates in 3D space, respectively, and $L_{pose}$ is the sum of the errors of the 21 hand joint point coordinates.

(17) $$L_{len}=\sum_{i,j}\left|{\left\|b_{i,j}\right\|}_{2}-{\left\|\overset{\wedge }{b_{i,j}}\right\|}_{2}\right|$$

(18) $$L_{dir}=\sum_{i,j}\left\|\frac{b_{i,j}}{{\left\|b_{i,j}\right\|}_{2}}-\frac{\overset{\wedge }{b_{i,j}}}{{\left\|\overset{\wedge }{b_{i,j}}\right\|}_{2}}\right\|$$

$L_{len}$ and $L_{dir}$ losses avoid distortion of the estimated obtained hand pose by imposing orientation and length constraints on the joints to provide a more rigid and natural hand skeleton structure.

Here, $b_{i,j}=\varphi_{i}-\varphi_{j}$ is the true bone vector between joints i and j, and $\overset{\wedge }{b_{i,j}}=\overset{\wedge }{\varphi_{i}}-\overset{\wedge }{\varphi_{j}}$ is the predicted bone vector estimated for the corresponding joint, $i,j\in \left[0,20\right]$.

## 4. Experiments

### 4.1. Experimental Environment

This study utilizes the publicly available STB dataset as its experimental dataset. STB is a dataset of single-hand real hand poses collected under six different backgrounds and varying lighting conditions. It comprises 18,000 color images with a resolution of 640 × 480 pixels, and the dataset provides 2D and 3D annotations of 21 key hand points, along with camera parameters. For this study, 15,000 images are selected as training samples, while 3000 images are used as test samples. STB is a one-handed real hand pose dataset collected under six different backgrounds and lighting conditions. In this study, 3000 randomly selected images are used to evaluate the experimental model in the dataset. This dataset mainly focuses on the position of each joint point in the hand and includes 10 gestures.

### 4.2. Experimental Settings

The experimental operating system for this study is Windows 7, and the programming language used is Python 3.6. The hardware development environment consists of an Intel(R) Core i7-6700 CPU @3.40GHz, 32GB of memory, and an NVIDIA GeForce GTX 1080 Ti GPU. This study utilizes the deep learning framework PyTorch 1.2 and CUDA version 10.0. The total number of training epochs is set to 400, with a batch size of 4. The RMSprop optimizer is employed, with an initial learning rate (lr) of 1 × 10^−4^. The learning rate is reduced to 10% of its original value every 50 epochs.

## 5. Results

### 5.1. Qualitative Comparison

**Figure 6:**
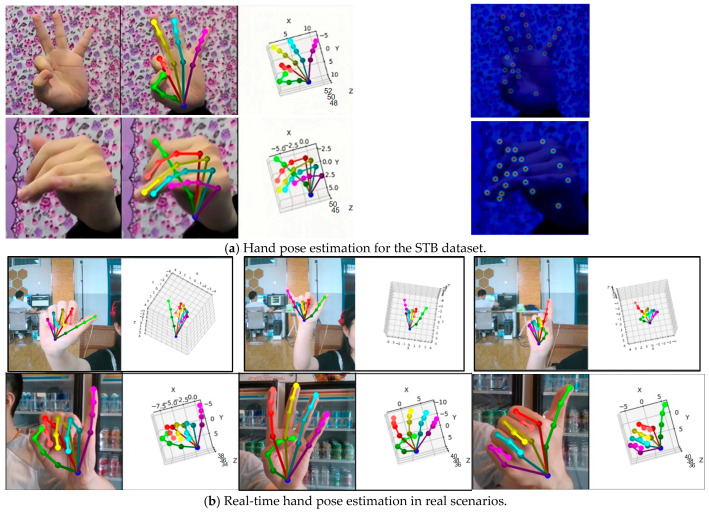
Hand pose estimation visualization results.

The visualization of the partial hand pose estimation results of our proposed method on the STB dataset and in real-world scenarios is shown in Figure 6. Subfigure (a) presents the hand pose estimation results on the STB dataset, while subfigure (b) displays the real-time hand pose estimation results in real-world scenarios. It can be observed that our proposed method achieves accurate estimation of hand joint positions on the STB dataset and in real-world scenarios. This is achieved by enhancing the features of each joint using the graph estimation module, which enables better estimation even in the presence of self-occluded joints.

### 5.2. Quantitative Comparison

#### 5.2.1. Comparative Experiments with Different Methods

**Figure 7:**
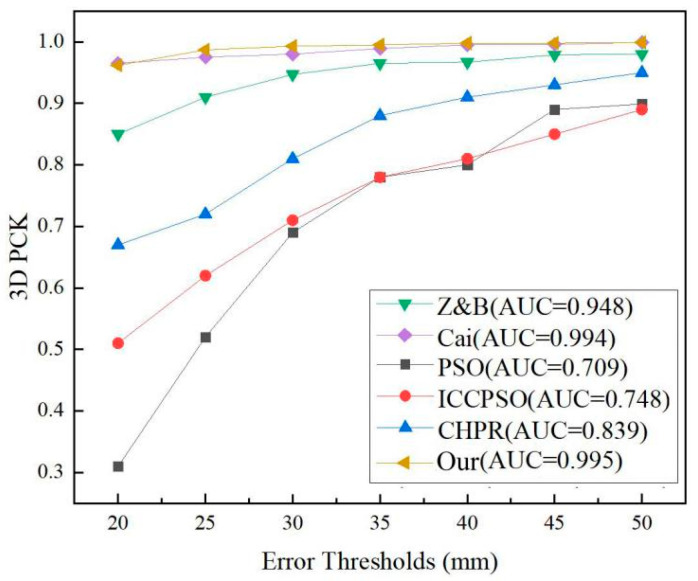
Comparison of the experimental results of different methods.

To evaluate the effectiveness of our proposed method, we compared it with other state-of-the-art hand pose estimation methods. Specifically, we compared the performance in terms of the 3D hand gesture joint estimation error (EPE), the proportion of correctly estimated hand gesture joints (PCK), and the area under the curve (AUC). The experimental results, which are presented in Table 1 and Figure 7, highlight the performance of our method in comparison to others, and the x-axis represents the error between the true coordinates of the hand joint points and the predicted coordinates of the model, measured in millimeters (mm). The error, EPE, in Table 1 is defined as the average Euclidean distance between the predicted 3D hand joint coordinates and the real 3D hand joint coordinates (GroundTruth). The formula is defined as follows:

(19) $$EPE=\sum_{i=1}^{N}\sqrt{{\left(x_{i}^{pre}-x_{i}^{gt}\right)}^{2}+{\left(y_{i}^{pre}-y_{i}^{gt}\right)}^{2}+{\left(z_{i}^{pre}-z_{i}^{gt}\right)}^{2}}$$

Here, *i* and *N* represent the sample number and total number of samples, ($x^{gt}$, $y^{gt}$, $z^{gt}$) are the real 3D pose coordinates in the dataset, and ($x^{pre}$, $y^{pre}$, $z^{pre}$) are the 3D pose coordinates estimated by the model. The average of three coordinates should be taken for each joint in 3D, and the estimated final result is a single value.

From the data in Table 1, it can be observed that our proposed method achieves lower EPE (mean) and EPE (median) values than other methods. This indicates that our method achieves smaller detection errors and more accurate localization of hand gesture joint positions. In terms of estimation accuracy, our method outperforms the methods proposed in [19,28] with improvements of 0.047 and 0.001, respectively. When the threshold error is taken as 0–50 mm, compared with Cai et al.’s method in reference [28], the AUC value of our method is increased by 0.001. Figure 7 illustrates the PCK curve variations in our proposed method and other methods within the threshold range of 0–50 mm. It can be clearly observed that our method outperforms the other methods, maintaining good accuracy even at smaller error thresholds. Overall, our proposed method demonstrates good stability.

#### 5.2.2. Ablation Experiment

In order to separately evaluate the effectiveness of the distribution-aware representation method for hand gesture joint coordinates, the graph reasoning module for hand gesture joint inference, and the bone constraint loss for hand pose estimation, this section presents our ablation experiments. Table 2 presents the experimental results of hand pose estimation by utilizing different node encoding and decoding methods based on the stacked hourglass model. Table 3 and Table 4 using the stacked hourglass model as the baseline, present the results of ablation experiments, carried out by incorporating the distribution-aware representation method for hand gesture joint coordinates, the graph reasoning module, and the bone constraint loss. The aim is to evaluate the impact of these improvements on the hand gesture joint estimation error and accuracy for both 2D and 3D hand pose estimation.

This study first evaluates the effectiveness of the distribution-aware representation method for hand gesture joint coordinates in 3D hand pose estimation. Various encoding and decoding methods are combined in the experiments. The encoding methods include biased and unbiased encoding. In biased encoding, the rounding operation is applied during the heatmap encoding process, while in unbiased encoding, the unbiased heatmaps are generated with non-quantized coordinates as the center. The decoding methods include unbiased decoding, standard offset decoding, and Taylor expansion offset decoding. In unbiased decoding, the hand gesture joint coordinates are obtained by selecting the position with the maximum activation in the heatmap. In standard offset decoding, the maximum activation value is offset by 0.25 pixels towards the second maximum activation value. The details are presented below.

From the data in Table 2, it can be observed that when using biased encoding, the decoding method based on Taylor expansion offset reduces the hand gesture joint errors, measured based on EPE (mean) and EPE (median), by 3.906 mm, 1.767 mm, 4.581 mm, and 2.442 mm, compared with unbiased decoding and standard offset decoding, respectively. Moreover, using unbiased encoding and the Taylor expansion offset decoding method results in the smallest error in hand gesture joints, with an AUC value of 0.785, which is a 14.77% improvement compared with the standard method. These experimental results demonstrate the effectiveness of the distribution-aware representation method in mitigating the encoding and decoding quantization errors for hand gesture joints.

From the data in Table 3, it can be observed that by using a stacked hourglass network as the baseline and incorporating a graph reasoning module for feature extraction and fusion after the first four-stage hourglass, the hand pose estimation error decreases, and the AUC value improves by 0.106. This demonstrates that the inclusion of graph convolutional networks can extract physical and structural features of the hand, enhancing the features of each hand gesture joint and improving the accuracy of the pose estimation. Furthermore, after incorporating the distribution-aware representation method in the encoding and decoding stages, the hand gesture joint error further decreases, and the AUC value improves by 0.103. These results indicate the effectiveness of reducing encoding and decoding errors and incorporating graph convolutional networks for 2D hand pose estimation.

According to the data in Table 4, it can be observed that by using a stacked hourglass network as the baseline and incorporating a graph reasoning module into the baseline network, the 3D hand pose estimation errors, measured based on EPE (mean) and EPE (median), decrease by 2.779 and 2.765, respectively, and the AUC value improves by 0.110. Furthermore, by using the distribution-aware representation method, the hand pose joint error decreases further, and the AUC value improves by 19.4%. Additionally, by applying a skeletal constraint loss function to impose constraints on joint lengths and orientations, the joint error, measured based on EPE (mean) and EPE (median), decreases by 1.40% and 2.99%, respectively, and the AUC value improves by 0.50%, reaching 0.995. These results demonstrate the effectiveness of the proposed improvements in reducing hand pose joint detection errors and enhancing estimation accuracy. Moreover, to verify the real-time performance of the proposed method, we conducted tests in a hardware development environment with an Intel (R) Core i7-6700 CPU @ 3.40GHz, 32GB of memory, and an NVIDIA GeForce GTX 1080 Ti GPU. The highest frame rate can reach 28 fps, which basically meets the usage requirements. In the future, we will study lighter models to meet higher real-time performance.

## 6. Discussion

In recent years, there has been rapid development in human–computer interaction technology, encompassing hardware device interaction, touch interaction, and motion recognition interaction, which has led to remarkable achievements in various domains. Gesture interaction, in particular, has garnered significant attention due to its naturalness during the interaction process. However, hand pose interaction poses considerable challenges due to the high degree of freedom of the human hand, potential self-occlusion between the fingers and the palm, and quantization errors in estimating key hand pose point coordinates from heatmaps. When using a stacked hourglass network for hand pose estimation, each key hand pose point coordinate is used to generate a heatmap as the mean of a Gaussian distribution function. This process introduces quantization errors in the heatmap generation, which, in turn, affect the precise localization of key hand pose points. In the process of 2D pose estimation, known as coordinate decoding, existing methods typically select the coordinates of the maximum activation position on the heatmap as the final result or perform manual shifting operations from the maximum activation to the second-highest activation direction, which also introduces errors. Therefore, this paper addresses the quantization errors in key point encoding and decoding by proposing a distribution-aware representation method to correct the errors. To consider the structural features of key hand pose points, a graph reasoning module based on graph convolutional networks and skeletal constraint loss is introduced. The proposed method, based on coordinate correction and graph convolution, aims to estimate 3D hand pose poses. The theoretical foundation of the proposed method is analyzed, and its effectiveness is experimentally validated.

This paper presents a method for estimating hand pose poses based on coordinate correction and graph convolution. The proposed method utilizes a distribution-aware representation of key point coordinates to generate unbiased heatmaps centered around non-quantized coordinates during the encoding process. Additionally, in the decoding process, the method considers the distribution information of heatmap activations thoroughly to achieve coordinate decoding based on Taylor expansion, thereby reducing errors in key point coordinate regression. Furthermore, the graph convolution module is employed to reconstruct the features of each key point through graph convolution, enabling better feature learning for each key point. By incorporating the distribution-aware representation of key point coordinates and the graph convolution module, the proposed method improves the accuracy of estimations of key hand pose points. Consequently, this method addresses the challenges of insufficient feature extraction of key hand pose points due to self-occlusion between fingers and the inaccuracies caused by quantization errors in the coordinate transformation process. Building upon a stacked hourglass network as the baseline model, the proposed method effectively corrects the errors in heatmap generation and regression of key point coordinates using the distribution-aware representation of key point coordinates. Additionally, a graph reasoning module is introduced after the hourglass network to model key hand pose points, reconstruct their features, and apply skeletal constraint loss to constrain the hand structure, further enhancing the accuracy of the hand pose estimation.

With the progress of computer vision, gesture interaction based on computer vision has received significant attention. Hand detection and hand pose estimation are key research areas within the field of gesture interaction. Hand detection involves identifying the hand region in an image or video, while hand pose estimation estimates the coordinates of key hand points based on hand detection. Subsequently, the corresponding hand pose is inferred from these coordinate relationships, enabling non-contact human–computer interaction and enhancing the user experience [32]. However, gesture interaction technology is still the subject of continuous research and exploration. Deep-learning-based hand pose estimation methods still face several challenges that warrant further investigation. Firstly, hand pose estimation based on single RGB images suffers from a high degree of ambiguity in mapping from 2D to 3D coordinates. Annotated datasets with 3D information in color images are time-consuming and labor-intensive to create. Therefore, future research can explore semi-supervised- and unsupervised-learning-based algorithms for hand pose estimation. Secondly, the proposed method in this paper focuses only on hand pose estimation for a single hand. However, human–computer interaction scenarios can be complex, and therefore, future research should investigate hand pose estimation for both hands and gestures involving interactions with objects.

## 7. Conclusions

To address the issue of self-occlusion between fingers in color-based hand pose estimation, this paper proposes a method for hand pose estimation based on coordinate correction and graph convolution. The method utilizes a stacked hourglass network as its base model and employs a distribution-aware coordinate representation for heatmap generation and error correction during heatmap regression to joint coordinates. Additionally, a graph reasoning module is introduced after the hourglass network to model the relationships between hand joints, reconstruct their features, and reduce translation and rotation errors using skeletal constraints. The experimental results demonstrate the effectiveness of the proposed method in improving the accuracy of hand pose estimation. However, it should be noted that this paper’s proposed hand pose estimation algorithm requires the construction of complex network models to enhance the estimation accuracy. In future research, lightweight hand pose estimation algorithms can be explored through techniques such as knowledge distillation and transfer learning to address these limitations.

## Figures and Tables

**Table 1:** Comparison of the results of different methods.

Algorithm	EPE (Mean)	EPE (Median)	AUC (0–50 mm)
Z&B [19]	12.210 mm	9.405 mm	0.948
Cai [28]	7.342 mm	4.561 mm	0.994
PSO [29]	-	-	0.709
ICCPSO [30]	-	-	0.748
CHPR [31]	-	-	0.839
Our	9.284 mm	5.178 mm	0.995

**Table 2:** Experimental results of 3D hand pose estimation under different encoding and decoding modes.

Encoding Method	Decoding Method	EPE (Mean)	EPE (Median)	AUC (0–50 mm)
Biased	No Offset	18.374 mm	16.137 mm	0.628
	Standard Offset	16.235 mm	13.998 mm	0.684
	Taylor Expanded Offset	14.468 mm	11.556 mm	0.731
Unbiased	Taylor Expanded Offset	13.949 mm	11.113 mm	0.785

**Table 3:** Results of 2D gesture posture evaluation and prediction ablation experiment.

Modeling	EPE (Mean)	EPE (Median)	AUC (0–50 mm)
Hourglass	13.346 mm	11.448 mm	0.789
Hourglass + Graph Reasoning	9.951 mm	8.274 mm	0.895
Hourglass + Graph Reasoning + DARK	6.895 mm	5.479 mm	0.998

**Table 4:** Results of 3D hand pose estimation ablation experiment.

Modeling	EPE (Mean)	EPE (Median)	AUC (0–50 mm)
Hourglass	16.235 mm	13.998 mm	0.684
Hourglass + Graph Reasoning	13.456 mm	11.233 mm	0.794
Hourglass + Graph Reasoning + DARK	10.782 mm	7.389 mm	0.948
Hourglass + Graph Reasoning + DARK+ Loss of Skeletal Restraint	9.284 mm	5.178 mm	0.995

## Data Availability

The data are contained within this article.
